# Nivolumab plus ipilimumab: a potential regimen to rewrite treatment guidelines for ESCC

**DOI:** 10.1038/s41392-022-01022-x

**Published:** 2022-05-25

**Authors:** Yuejun Luo, Nan Sun, Jie He

**Affiliations:** 1 Department of Thoracic Surgery, National Cancer Center/National Clinical Research Center for Cancer/Cancer Hospital, Chinese Academy of Medical Sciences and Peking Union Medical College, 100021 Beijing, China; 2 State Key Laboratory of Molecular Oncology, National Cancer Center/National Clinical Research Center for Cancer/Cancer Hospital, Chinese Academy of Medical Sciences and Peking Union Medical College, Beijing, China

**Keywords:** Gastrointestinal cancer, Tumour immunology

The recent research published in *The New England Journal of Medicine* by Y. Doki et al. has reported the interim findings from the CheckMate 648, which is an international, multi-center, open-label, and randomized phase 3 clinical trial to explore the role of dual immune checkpoints inhibitors combination for patients with advanced esophageal squamous cell carcinoma (ESCC)^1^.

This study evaluated the efficacy and safety of the combination of PD-1 and CTLA-4 inhibitors for patients with advanced ESCC, aiming to provide some enlightenment for advanced ESCC treatment.

Patients recruited in the CheckMate 648 were diagnosed with advanced, metastatic, or recurrent ESCC. The details of enrollment criterion included age more than 18-year, naïve to systemic therapy for advanced disease, measurable disease, pathological identification of ESCC or esophageal adenosquamous carcinoma. All 970 patients were randomly divided into nivolumab plus chemotherapy, nivolumab plus ipilimumab, and chemotherapy alone groups for 1:1:1. The primary endpoints included progression-free survival (PFS) and overall survival (OS), while objective response rate was the secondary endpoint. Compared with the chemotherapy alone group (median PFS: 5.6 months, median OS: 10.7 months), the nivolumab plus chemotherapy group demonstrated longer PFS (median PFS: 5.8 months) and OS (median OS: 13.2 months) in all populations, and the nivolumab plus ipilimumab groups exhibited favorable OS (median OS: 12.7 months) in all populations, however, only patients with PD-L1 expression ≥1% achieved significantly longer median duration of response than chemotherapy group (11.8 months vs. 5.7 months). For safety, the occurrence of grade 3 and higher-level treatment-related adverse was 36%, 47%, and 32% in the chemotherapy alone, nivolumab plus chemotherapy, and nivolumab plus ipilimumab groups, respectively. The combination group suggested acceptable toxicity for participants.

In China, ESCC is one of the most common digestive tumors, accounting for about 90% of cases worldwide. Most patients have progressed to advanced disease by the time of diagnosis, losing the opportunity for surgical resection, with an unfavorable survival. The monotherapy combined with chemotherapy achieved significant survival benefits for patients with ESCC^2^. However, the adverse effects related to chemotherapy are difficult matters in clinical. The dual immune checkpoints inhibitors regimen exhibited synergistic anti-tumor effects in cancers, with higher objective response rates and better clinical outcomes than PD-1 inhibitor alone, as well as a manageable toxicity profile (Table 1). Researchers are thinking about whether this regimen can be used in advanced ESCC for the purpose of de-chemotherapy. In this study, the role of the nivolumab plus ipilimumab regimen was firstly explored in advanced ESCC treatment. Compared to previous dual inhibitors trials involved in esophageal cancer (Checkmate 032 of metastatic esophagogastric cohort), this study enrolled a considerable number of participants and provided more reliable evidence to support the potential efficacy of this dual inhibitors combination, which is also consistent with the results of Checkmate 032. This study also achieved comprehensive subgroup analyses to identify the potential biomarker (PD-L1) of response to this dual inhibitors regimen. The study exhibited promising results, especially in patients with PD-L1 expression ≥1%. Unfortunately, patients with negative PD-L1 expression seemed to benefit little from this combination, and a constant follow-up is needed to observe whether patients with negative PD-L1 will eventually benefit from this regimen. Therefore, chemotherapy still has a proper place in the treatment of advanced ESCC, especially PD-1 inhibitor combined with chemotherapy is the most widely applied treatment. It is worthwhile to consider whether the combination of chemotherapy with dual inhibitors can enhance the responses of patients, especially those patients with negative PD-L1, with manageable toxicities.

**Table 1:** The summary of published clinical trials of nivolumab plus ipilimumab and nivolumab monotherapy in various tumors

Cancer type	Year	Patients	Therapy	Primary outcomes	Grade 3–4 adverse events rate	Treatment-related deaths
Esophagogastric cancer (Checkmate 032)	2018	160	N 3mg/Kg	ORR: 12% (95 CI%: 5–23%)	17%	0/59
			N 1mg/Kg plus I 3mg/Kg	ORR: 24% (95 CI%: 13–39%)	47%	0/49
			N 3mg/Kg plus I 1mg/Kg	ORR: 8% (95 CI%: 2–19%)	27%	1/52
mUC (Checkmate 032)	2019	274	N 3mg/Kg	ORR: 25.6% (95 CI%: 16.4–36.8%)	26.9%	1/78
			N 1mg/Kg plus I 3mg/Kg	ORR: 38% (95 CI%: 28.1–48.8%)	39.1%	0/92
			N 3mg/Kg plus I 1mg/Kg	ORR: 26.9% (95 CI%: 18.7–36.5%)	30.8%	1/104
SCLC (Checkmate 032)	2020	243	N	ORR: 11.6% (95 CI%: 6.9–17.9%)	12.9%	1/147
			N plus I	ORR: 21.9% (95 CI%: 14.1–31.5%)	37.5%	3/96
mCRC (Checkmate 142)	2018	193	N	ORR: 31% (95 CI%: 20.8–42.9%)	20.3%	0/74
			N plus I	ORR: 55% (95 CI%: 45.2–63.8%)	32%	0/119
Melanoma (Checkmate 067)	2021	945	N	OS: 36.9 months (95 CI%: 28.2–58.7)	22%	1/313
			I	OS: 19.9 months (95 CI%: 16.8–24.6)	28%	1/311
			N plus I	OS: 72.1 months (95 CI%: 38.2–not reached)	59%	2/313
OCSCC (NCT02919683)	2020	29	N	MPR rate: 7.1% (1/14)	14.3%	0/14
			N plus I	MPR rate: 20% (3/15)	33.3%	0/15
NSCLC (NCT03158129)	2021	44	N	MPR rate: 22% (5/23)	13%	1/23
			N plus I	MPR rate: 38% (8/21)	10%	0/21
EOC (NCT02498600)	2020	100	N	ORR: 12.2%	33%	0/49
			N plus I	ORR: 31.4%	49%	0/51
MPM (NCT02716272)	2021	125	N	12-week DCR: 39.7% (95 CI%: 27.6–51.8%)	14.3%	0/63
			N plus I	12-week DCR: 51.6% (95 CI%: 39.2–64.1%)	26.2%	3/61
Metastatic sarcoma (Alliance A091401)	2018	85	N	ORR: 5% (92 CI%: 1–15%)	7%	0/43
			N plus I	ORR: 16% (92 CI%: 7–29%)	14%	0/42

Notes: *N* Nivolumab, *I* Ipilimumab, *ORR* Objective response rate, *mUC Metastatic urothelial carcinoma, SCLC* Small cell lung cancer, *mCRC* Metastatic colorectal cancer, *OS* Overall survival, *OCSCC* Squamous cell carcinoma of the oral cavity, *MPR* Major pathologic response, *NSCLC* Non-small cell lung cancer, *EOC* Epithelial ovarian cancer, *MPM* Malignant pleural mesothelioma, *DCR* Disease control rate

The authors failed to explore more subgroups analyses except for PD-L1. Previous studies have observed patients with blood-Tumor burden mutation (bTMB) ≥20 mut/Mb or high TMB regardless of PD-L1 expression significantly benefited from the PD-L1 plus CTLA-4 inhibitors in lung cancer^3,4^. In metastatic colorectal cancer, patients with microsatellite instability-high/mismatch repair-deficient (MSI-H/dMMR) demonstrated a durable clinical benefit from this dual inhibitors regimen^5^. We speculated that bTMB, TMB, and MSI-H/dMMR may be potential biomarkers of this combination in advanced ESCC. The robust biomarkers are warranted to select patients sensitive to dual inhibitors, maximizing their survival benefits and avoiding serious adverse effects of chemotherapy. Moreover, whether this dual immune checkpoint blockades regimen can be applied to the other treatment period of ESCC, for example, as neoadjuvant therapy to improve pathological complete rates for patients with PD-L1 expression ≥1%, or higher bTMB and TMB, or MSI-H/dMMR?

Novel anti-tumor drugs are being vigorously developed. In the future, we believe that newly developed drugs may be available in combination with current immune checkpoint inhibitors to help patients with ESCC achieve a favorable prognosis and quality of life without chemotherapy. To improve the outcome of patients with advanced ESCC, we need to take a two-pronged approach. Firstly, we need to examine the role of potential therapy regimens in the efficacy of ESCC with a view to finding the most promising treatment modalities. Secondly, since the tumor microenvironment varies greatly among patients with ESCC, the same treatment suggests different efficacy in different patients. It is essential to select individualized treatments for every patient. Whether it is an immunotherapy plus chemotherapy regimen, a dual immunotherapy combination, or a novel option in the future, how to apply these regimens to the appropriate patients is a major concern. The robust biomarkers are warranted to help us screen out the most appropriate patients for different regimens, which achieve a favorable prognosis and avoid unnecessary side effects for patients with ESCC. The patient’s physical tolerance and contraindications to treatments should also be considered. Personalized treatment and management have been the primary clinical concern for ESCC, which needs to be continuously improved.

Improving the clinical outcomes of advanced ESCC has been a formidable challenge. The results of CheckMate 648 comprehensively uncovered the function of nivolumab plus ipilimumab combination in advanced ESCC, which is a novel attempt at the de-chemotherapy regimen. Although this dual immunotherapy did not suggest very promising achievements in all populations, it still has favorable efficacy in patients with PD-L1 expression ≥1%. The findings provided valuable insights into the clinical management of advanced ESCC.
